# Pediatric intensive care unit treatment alters the diversity and composition of the gut microbiota and antimicrobial resistance gene expression in critically ill children

**DOI:** 10.3389/fmicb.2023.1237993

**Published:** 2023-11-13

**Authors:** Jiayue Xu, Xiangmei Kong, Jiru Li, Haoyun Mao, Yueniu Zhu, Xiaodong Zhu, Yaya Xu

**Affiliations:** Department of Pediatric Intensive Care Medicine, Xinhua Hospital, Affiliated to the Medical School, Shanghai Jiao Tong University, Shanghai, China

**Keywords:** gut microbiota, critical illness, children, infection, drug resistance genes

## Abstract

**Introduction:**

Common critical illnesses are a growing economic burden on healthcare worldwide. However, therapies targeting the gut microbiota for critical illnesses have not been developed on a large scale. This study aimed to investigate the changes in the characteristics of the gut microbiota in critically ill children after short-term pediatric intensive care unit (PICU) treatments.

**Methods:**

Anal swab samples were prospectively collected from March 2021 to March 2022 from children admitted to the PICU of Xinhua Hospital who received broad-spectrum antibiotics on days 1 (the D1 group) and 7 (the D7 group) of the PICU treatment. The structural and functional characteristics of the gut microbiota of critically ill children were explored using metagenomic next-generation sequencing (mNGS) technology, and a comparative analysis of samples from D1 and D7 was conducted.

**Results:**

After 7 days of PICU admission, a significant decrease was noted in the richness of the gut microbiota in critically ill children, while the bacterial diversity and the community structure between groups remained stable to some extent. The relative abundance of *Bacilli* and *Lactobacillales* was significantly higher, and that of *Campylobacter hominis* was significantly lower in the D7 group than in the D1 group. The random forest model revealed that *Prevotella coporis* and *Enterobacter cloacae* were bacterial biomarkers between groups. LEfSe revealed that two Gene Ontology entries, GO:0071555 (cell wall organization) and GO:005508 (transmembrane transport), changed significantly after the short-term treatment in the PICU. In addition, 30 KEGG pathways were mainly related to the activity of enzymes and proteins during the processes of metabolism, DNA catabolism and repair, and substance transport. Finally, 31 antimicrobial resistance genes had significantly different levels between the D7 and D1 groups. The top 10 up-regulated genes were Erm(A), ErmX, LptD, eptB, SAT-4, tetO, adeJ, adeF, APH(3′)-IIIa, and tetM.

**Conclusion:**

The composition, gene function, and resistance genes of gut microbiota of critically ill children can change significantly after short PICU treatments. Our findings provide a substantial basis for a better understanding of the structure and function of gut microbiota and their role in critical illnesses.

## 1. Introduction

Common critical illnesses, including sepsis, acute respiratory distress syndrome, and multiorgan failure, are a growing economic burden on healthcare worldwide (Adhikari et al., 2010). Pathophysiological changes that occur in critically ill patients can affect the composition of the intestinal flora. For example, both the hypoperfused and reperfused states of the intestinal wall can lead to intense mucosal inflammation, which further leads to changes in the intestinal environment. Increased nitrate concentrations and altered mucosal oxygen concentrations favor the growth of *Aspergillus phylum* microorganisms, including clinically common gram-negative *Bacilli* such as *Pseudomonas aeruginosa* and *Escherichia coli*. The growth of Firmicutes microorganisms, such as *Staphylococcus aureus* and *Enterococcus*, can also be affected (Dickson, 2016). Disturbances in the intestinal flora are associated with disease progression and clinical prognosis in critically ill patients. Ojima et al. (2022) showed that dysbiosis in the ratio of the *Bacteroides* to the Firmicutes (B/*F* > 8 or B/*F* < 1/8) in critically ill patients within 7 days of admission was associated with increased mortality. Andremont et al. (2020) also showed that carrying rectal or pharyngeal ultra-broad-spectrum β-lactamase-producing *Enterobacteriaceae* was a risk factor for the subsequent development of ventilator-associated pneumonia caused by bacterial infection in critically ill patients. In addition, a strong link exists between the intestinal flora and function of organs such as the brain, lungs, liver, and kidneys. In a study by Dickson et al. (2020) intestine-associated bacteria such as *Trichophytonaceae* and *Enterobacteriaceae* were present in the pulmonary flora of critically ill patients, and an increase in these bacteria predicted the number of days a patient would have without being on a ventilator. The loss of endothelial barrier integrity and dysbiosis of the intestinal flora are major pathophysiological alterations in sepsis that play important roles in sepsis-related acute kidney injury (Xu et al., 2022).

Most bacterial communities in the gut have a symbiotic relationship with the human host; however, many conditionally pathogenic bacteria, such as *Enterobacteriaceae* and *Enterococcaceae*, are also present in the gut and can cause severe infections in immunocompromised patients (Mcinnes et al., 2020). Antibiotic use is a key factor in the induction of drug-resistant genes. In recent years, infections caused by drug-resistant strains of *Escherichia coli*, *Klebsiella pneumoniae*, and *Enterococcus faecalis* have been increasing worldwide (Guzman Prieto et al., 2016; Dunn et al., 2019). In addition, some studies have shown that the expression levels of resistance genes in the gut microbiota increase during antibiotic treatment in children; however, not all resistance genes return to baseline levels after antibiotic discontinuation (Yassour et al., 2016).

Therefore, gut microbiota has gathered increasing attention from clinicians as an important element in the management of critically ill patients. Despite decades of medical and technological advances, therapies targeting the gut flora for critical illnesses have not been developed on a large scale. Metagenomics next generation sequencing (mNGS) is a new method of high-throughput RNA/DNA sequencing that enables the detection of all pathogenic bacteria in a sample without bias (Diao et al., 2022; Gökdemir et al., 2022). This method allows further exploration of the composition, diversity, gene function, and drug resistance genes of the gut microbiota.

This study aimed to describe the changes in the composition, gene function, and drug resistance genes of gut microbiota in critically ill children after receiving short-term PICU treatment and to provide a theoretical basis for the rational use of medication and reduction of the incidence of adverse events in PICU patients.

## 2. Materials and methods

### 2.1. Patients and sample collection

This study was approved by the Ethics Committee of Xinhua Hospital, Shanghai Jiao Tong University School of Medicine (approval number: XHEC-D-2022-255). This was a prospective observational study on children admitted to the PICU of Xinhua Hospital, affiliated with Shanghai Jiao Tong University School of Medicine, from March 2021 to March 2022 and who received broad-spectrum antibiotic therapy. The inclusion criteria were as follows: ① age > 28 days and < 18 years and ② receiving broad-spectrum antibiotics within the first 24 h of admission to the PICU. The exclusion criteria were: ① post-rectal surgery; ② presence of infection at the anal site; ③ no follow-up due to death or discharge within 7 days; ④ failure to obtain anal swab samples; and ⑤ refusal to participate in the study by the child’s guardian. Anal swabs were collected on days 1 (D1) and 7 (D7) of admission to the PICU from children who met the enrollment criteria. Standardized anal swab samples were collected: the children were placed in the lateral position, and a disposable sterile anal swab was used to enter the anus of the children approximately 3 cm deep, rotated for 2–3 s, and then removed and placed in a DNA preservation solution. The samples were transferred to a −80°C refrigerator for freezing within 24 h after the sampling.

### 2.2. DNA extraction and library preparation

Fecal DNA was extracted using the TIANamp Magnetic DNA Kit (Tiangen) according to the manufacturer’s instructions. The quantity and quality of DNA were assessed using the Qubit (Thermo Fisher Scientific) and NanoDrop (Thermo Fisher Scientific), respectively. DNA libraries were prepared using the Hieff NGS C130P2 OnePot II DNA Library Prep Kit for MGI (Yeasen Biotechnology) according to the manufacturer’s protocols. Agilent 2100 (Ronchetti et al., 2022) was used for quality control, and MGISEQ-2000 was used to sequence DNA libraries with single-end 50 bp tags (Lang et al., 2021).

### 2.3. Metagenomic next-generation sequencing

Before analysis, raw sequencing data were split using bcl2fastq2 (version 2.20), and high-quality sequencing data were generated using Trimmomatic (version 0.36) by removing low-quality reads, adapter contamination, duplicates, and short (length < 36 bp) reads. The human host sequence was subtracted by mapping to the human reference genome (hs37d5) using bowtie2 (version 2.2.6). Reads that could not be mapped to the human genome were retained and aligned with the microorganism genome database for microbial identification by Kraken (version 2.0.7) and estimated for species abundance using Bracken (version 2.5.0). The remaining genomes were compared to those in the microbial genome databases, including the genomes of bacteria, fungi, viruses, and parasites downloaded from GenBank version 238.^1^ Gut microbiota data were downloaded from the database of human gut microbiota,^2^ and all annotated results (OTUs) were compared with public data. Microorganisms with species detection rates >1% and mean abundances >0.001 were retained. mNGS was performed on an Illumina NovaSeq 6000 (Illumina) using a 150-bp paired-end read protocol.

### 2.4. Statistical analysis

Statistical analysis of the gut microbiota was performed using R software (version 3.6.0). Alpha diversity was estimated based on the taxonomic profile of each sample, and beta diversity was assessed by the Bray–Curtis and Jaccard–Curtis distances. PERMANOVA was performed using the R package vegan (Hu and Satten, 2022) to analyze Bray–Curtis and Jaccard–Curtis distances between groups, which were subsequently visualized using principal components analysis (PCA) and non-metric multidimensional scaling (NMDS). The relative abundance of microorganisms at different levels between groups was tested by the R package Kruskal Test (Zhang et al., 2021). The genera with mean relative abundances greater than 1% and penetrance greater than 40% among all samples were compared, and false discovery rate correction was adopted to adjust all *p* values. Accuracy and Gini indices were used to evaluate bacterial biomarkers between groups by the Random Forest predictive algorithm (Hu and Szymczak, 2023). Gene function prediction was performed based on our in-house and Human Project Unified Metabolic Analysis Network 2 (HUMAnN2) (Hu et al., 2022). Gene Ontology (GO) entries between groups were assessed using the linear discriminant analysis (LDA) of effect size (LEfSe) (Erawijantari et al., 2020). Those with an LDA score > 2.0 were considered biomarkers between groups. In addition, STAMP software was used to compare and visualize the different GO entries and KEGG pathways, with both having a corrected *p* value <0.01. According to the GO database^3^ and Kyoto Encyclopedia of Genes and Genomes (KEGG)^4^ database, gene functional characteristics were finally described. The comparison of drug resistance genes between groups was performed using DEseq2, and the statistical difference was set at *P* adjust <0.05 and |log2FC| > 1. These resistance genes were then described with reference to the Comprehensive Antibiotic Research Database (CARD; card.mcmaster.ca). Clinical data analysis was performed using the SPSS (v.25.0) software. The paired *t*-test or Wilcoxon signed-rank test was used for each continuous variable between the groups.

## 3. Results

### 3.1. Patient characteristics

**Figure 1:**
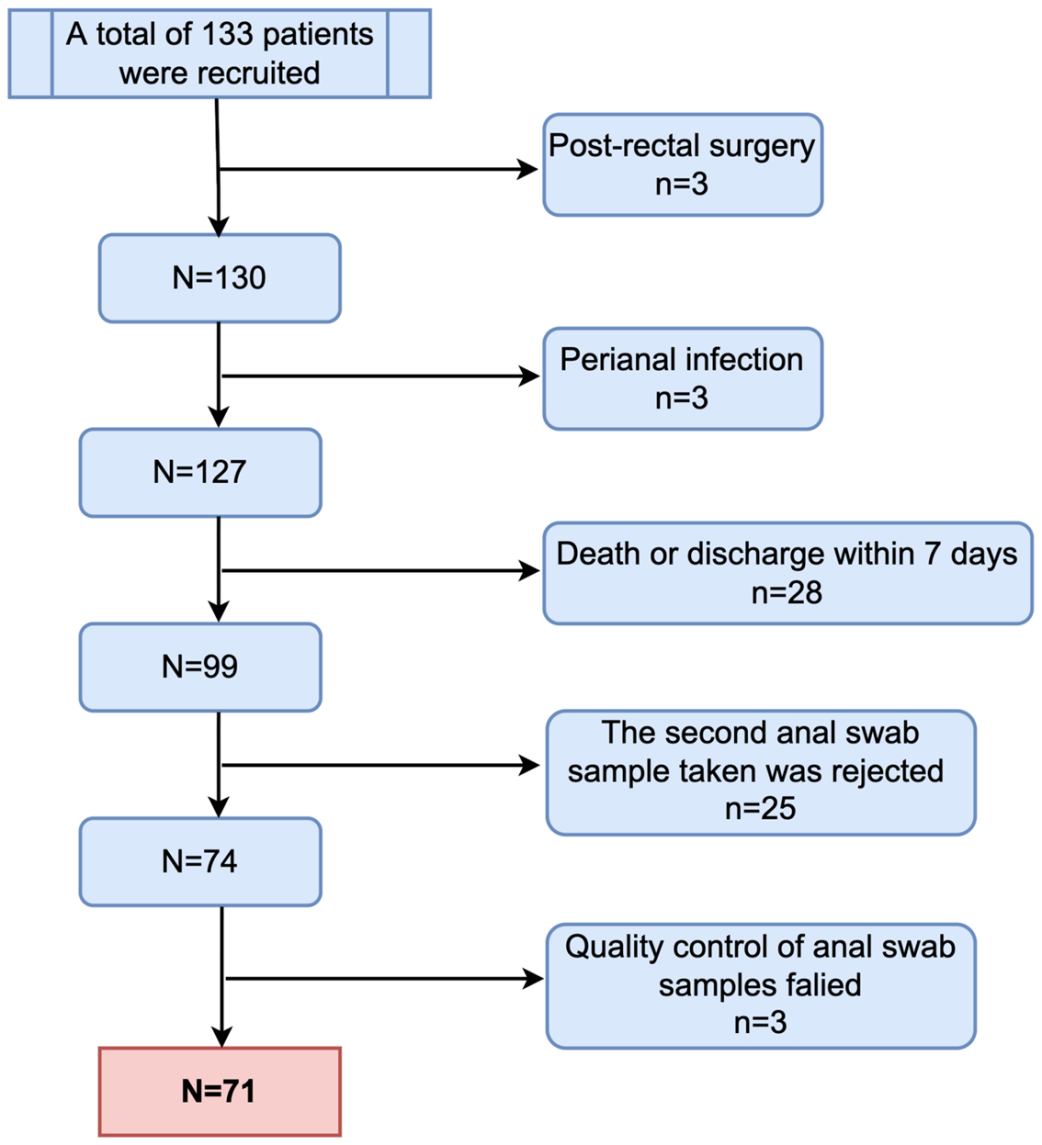
Flowchart of patient enrollment.

From March 2021 to March 2022, a total of 71 patients were enrolled in the study (Figure 1). Thirty-six of the included children were male (50.7%). Forty-one children were aged over 3 years (57.7%). Most of the enrolled children (71.4%) had underlying diseases, with malignant tumors or leukemia being the most common (19.7%), followed by neurological diseases (12.7%). The site of infection was identified based on the clinical presentation of the children and pathogenic culture results after admission. The respiratory system was the most common site of infection in the enrolled children, followed by the central nervous system, bloodstream infections, urinary system, digestive system, and skin and soft tissues in that order, with approximately one-fifth of the children not having a clear site of infection. Additionally, 25 children had respiratory failure at the time of PICU admission, 10 had shock, 7 had renal insufficiency, 4 had disseminated intravascular coagulation (DIC), and 3 had hepatic insufficiency (Table 1).

**Table 1:** Patient characteristics.

Characteristic	Number of patients (*n* = 71)	Percentage (%)
Sex M/F	36/35	50.7/49.3
Age		
1 month–3 years	30	42.3
> 3 y	41	57.7
Weight (*Z*-score)		
<−2	11	15.5
−2 to 2	52	73.2
>2	8	11.3
Underlying disease		
None	21	29.6
Malignancy or leukemia	14	19.7
Inherited metabolic diseases	9	12.7
Congenital digestive tract malformation	6	8.5
Neurological diseases	13	18.3
Post-traumatic or surgical	7	9.9
Congenital heart disease	1	1.4
Infection site		
Respiratory	24	33.8
Blood	5	7
Gastrointestinal	2	2.8
Central nervous system	8	11.3
Urinary	4	5.6
Skin	1	1.4
Multi-site infection	12	16.9
Unspecified	15	21.1
Complications		
Shock	10	14.08
Respiratory failure	25	33.8
Acute kidney injury	7	9.86
Hepatic failure	3	4.23
DIC	4	5.63

### 3.2. Treatments of patients admitted to the PICU

All the children received intravenous antibiotic therapy upon admission. More than half of the children received two or more classes of antibiotics within 7 days of PICU admission, including antibiotic escalation, antibiotic de-escalation therapy, and antibiotic combinations. Oxazolidinone antibiotics, represented by linezolid, and glycopeptide antibiotics, represented by vancomycin, were the two antibiotics commonly used in the enrolled children, accounting for 78.9 and 74.6% of the total number of patients, respectively. Cefepime, a fourth-generation cephalosporin, was one of the main anti-infective drugs used by the enrolled children, accounting for 73.2% of the total number of users. More than one-third of the children received carbapenem-based antibiotics (Table 2).

**Table 2:** Use of antibiotics in critically ill children admitted to the PICU from March 2021 to March 2022.

Antibiotic use	*N*	Percentage (%)
Antibiotic type		
Fourth generation cephalosporins	52	73.2
Glycopeptide antibiotics	53	74.6
Carbapenem antibiotics	25	36.6
Oxazolidinone antibiotics	56	78.9
Combined antibiotics	38	53.5

Additionally, 22.5% of the children received proton pump inhibitors on day 1 of admission, 12% received vasoactive drugs, 22% were treated with invasive mechanical ventilation, 36.6% were treated with parenteral nutrition, and only a minority (2%) received probiotics. Although the proportion of children receiving supportive treatment changed on day 7 of admission, there was no statistically significant difference (Table 3).

**Table 3:** Clinical information of critically ill children admitted to the PICU from March 2021 to March 2022.

Clinical characteristics	D1	D7	*p*-value
Clinical indicators			
CRP (mg/L, P50 [P25, P75])	56.00 (4.00, 129.00)	5.00 (1.00, 22.00)	< 0.01
PCT (ng/mL, P50 [P25, P75])	0.69 (0.10, 5.17)	0.27 (0.09, 1.03)	< 0.01
WBC (×10^^9^/L, P50 [P25, P75])	11.41 (6.43, 15.91)	9.06 (5.77, 13.17)	0.01
Hb (g/L, P50 [P25, P75])	99.00 (84.00, 114.00)	93.00 (83.00, 107.00)	0.12
PLT (×10^^9^/L, P50 [P25, P75])	264 (142, 398)	310 (183, 425)	0.01
Creatine (μmol/L, P50 [P25, P75])	25.20 (18.2, 34.90)	20.40 (14.80, 30)	< 0.01
Bilirubin (mg/dL, P50 [P25, P75])	10.90 (6.70, 16.90)	9.20 (6.20, 16.90)	0.29
Albumin (g/L, P50 [P25, P75])	36.00 (30.40, 41.20)	37.50 (34.00, 39.70)	0.09
Lactate (mmol/L, P50 [P25, P75])	2.00 (1.50, 3.30)	1.70 (1.20, 2.30)	< 0.01
ICU treatments			
Proton pump inhibitor [*n*, (%)]	16 (22.5)	16 (22.5)	1.00
Vasoactive drugs [*n*, (%)]	12 (16.9)	14 (19.7)	0.66
Mechanical ventilation [*n*, (%)]	22 (31.0)	21 (29.6)	0.86
Parenteral nutrition [*n*, (%)]	26 (36.6)	17 (23.9)	0.10
Probiotic therapy [*n*, (%)]	2 (2.8)	6 (8.5)	0.15
Clinical outcomes			
28-day mortality [*n*, (%)]	7 (9.90)	/	/
PICU length of stay (day, P50 [P25, P75])	9.00 (5.00, 17.00)	/	/
Length of hospital stay (day, P50 [P25, P75])	26.00 (13.00, 45.00)	/	/

Data are presented as median (P50), 25th percentile (P25), and 75th percentile (P75) or number (*n*) and percentage (%). CRP, C-reactive protein; PCT, procalcitonin; WBC, white blood cell count; PLT, platelet count; PICU, pediatric intensive care unit.

Seven days after PICU treatment, a significant decrease as observed in the levels of C-reactive protein (*p* < 0.01), procalcitonin (*p* < 0.01), creatinine (*p* < 0.01), and lactate (*p* = 0.02) as well as white blood cell count (*p* = 0.01). A significant increase in platelet count (*p* = 0.01) was noted, compared with the admission levels. No significant changes were observed in the indicators, such as hemoglobin and liver function (total bilirubin and albumin). In terms of clinical outcomes, 7 children died during the 28-day follow-up period. The mean ± standard deviation of the length of stay in the PICU and the total length of hospital stay were 16.39 ± 28.06 and 36.55 ± 38.67 days, respectively (Table 3).

### 3.3. Changes in gut microbiome in critically ill children after short-term PICU treatment

#### 3.3.1. Short-term PICU treatment alters the richness of the gut microbiome but not the diversity and the community structure

**Figure 2:**
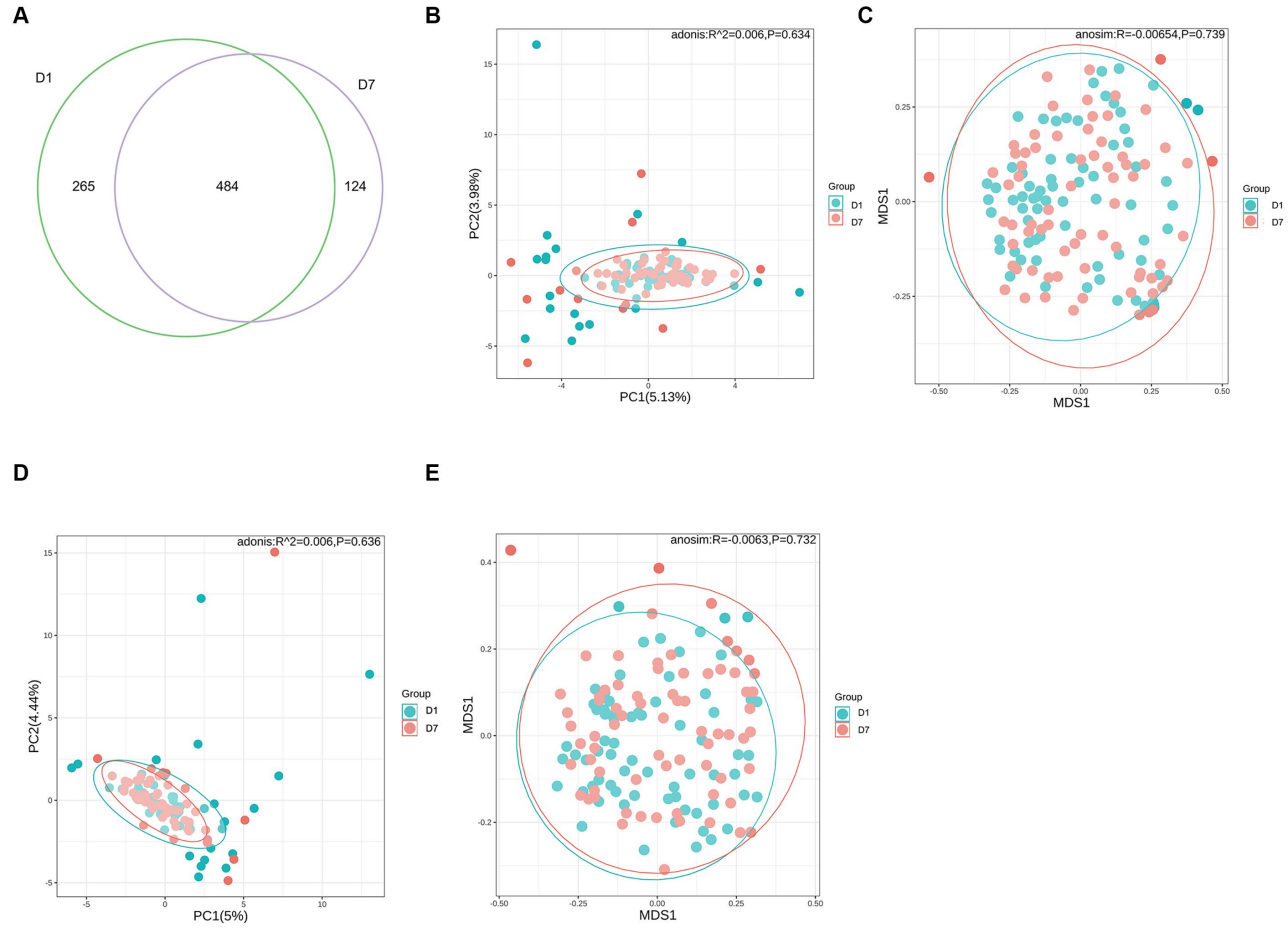
OTUs and β-diversity analysis of the microbiota in the D1 and D7 groups. **(A)** Venn diagram of the number of OTUs in the gut microbiota of children in groups D1 and D7. **(B–E)** PCA and NMDS analyses of β-diversity.

Based on the Wayne diagram of operational taxonomic units (OTU) (Figure 2A) at the species level, children had 749 OTUs on day 1 of PICU admission and 608 OTUs on day 7, of which, 484 OTUs were common to both groups. The species composition of the gut microbiota of critically ill children changed to some extent after a short treatment in the PICU.

Alpha diversity analysis was based on Shannon, Simpson, Chao1, and ACE indicators. As shown in Table 4, Shannon and Simpson indices in the D1 and D7 groups, which represent bacterial diversity, were 1.51 ± 0.80 vs. 1.38 ± 0.70 and 0.60 ± 0.28 vs. 0.57 ± 0.26 (both *p* > 0.05), respectively. The ACE and Chao1 indices in the D1 and D7 groups were 60.22 ± 68.10 vs. 40.62 ± 41.89 and 59.62 ± 67.50 vs. 40.62 ± 41.87 (both *p* < 0.01), indicating that the bacterial richness was significantly decreased in the D7 group (more detailed information can be found in Supplementary Table S1). These results showed that compared with that in the D1 group, the bacterial richness decreased significantly but not the evenness in the D7 group. The PCA and NMDS based on the Bray–Curtis and Jaccard-Curtis distances showed that the beta diversity of the gut microbiota did not differ significantly between the two groups (*p* > 0.05) (Figures 2B–E; Supplementary Table S2). Our results suggested that the community structure of the gut microbiome of the children remained somewhat stable even under the influence of antibiotics and other treatments they received.

**Table 4:** Comparison of α diversity between the D1 and D7 groups presented as median (P50), 25th percentile (P25), and 75th percentile (P75).

	D1 Group	D7 Group	*p*-value
Shannon index, P50 (P25, P75)	1.62 (0.88, 2.18)	1.46 (0.81, 1.91)	0.16
Simpson index, P50 (P25, P75)	0.72 (0.45, 0.82)	0.65 (0.43, 0.79)	0.2
ACE index, P50 (P25, P75)	32.00 (20.00, 54.00)	24.00 (20.00, 40.50)	<0.01
Chao1 index, P50 (P25, P75)	32.00 (20.00, 54.00)	24.00 (20.00, 40.50)	<0.01

#### 3.3.2. Changes in the relative abundance of gut microbiota community

**Figure 3:**
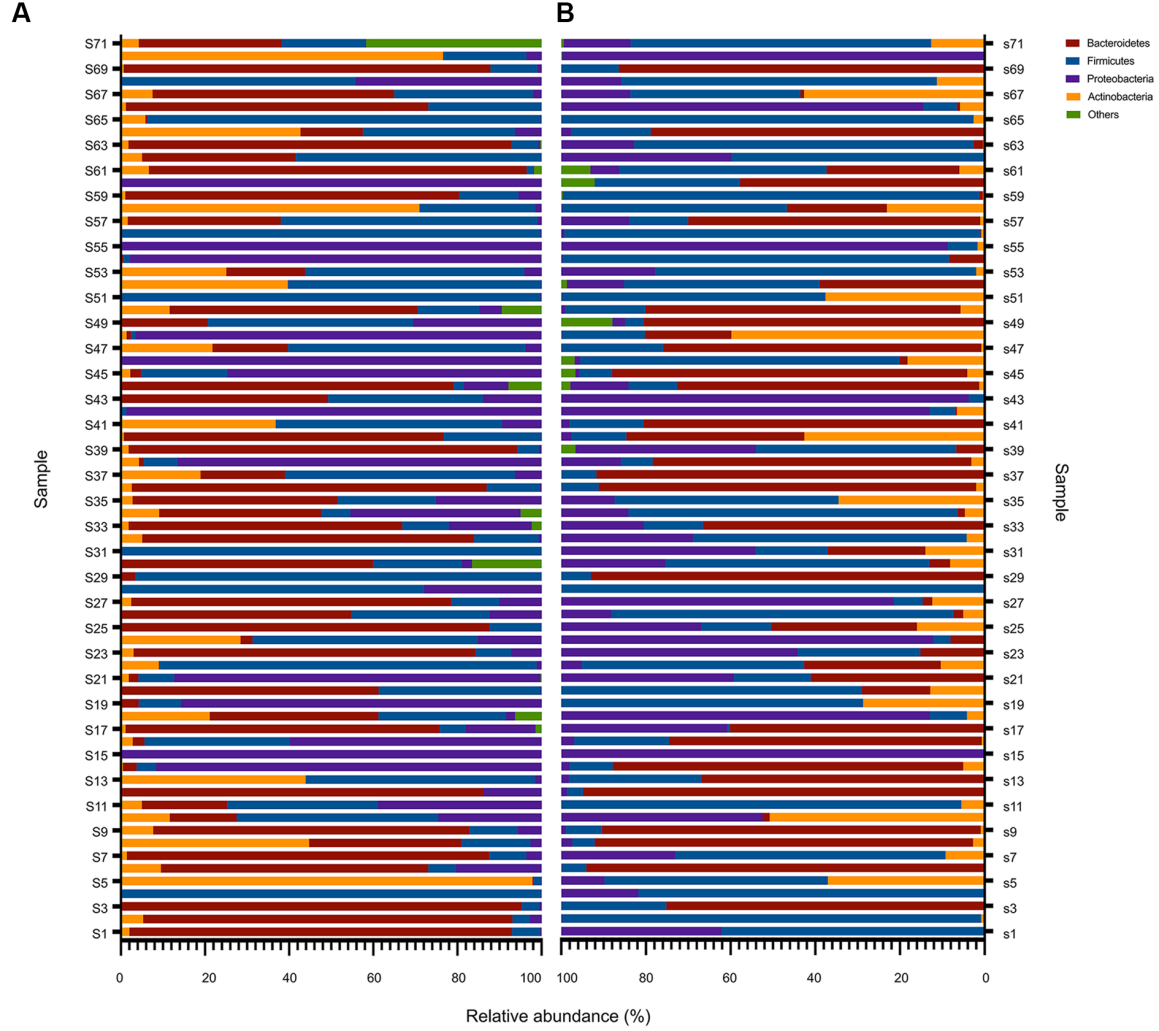
Overall composition of the bacteria at the phylum level before and after PICU treatments in critically ill children. Changes in the relative abundance of bacteria at the phylum level in the gut microbiota of critically ill children in the D1 **(A)** and D7 **(B)** groups. Based on the metagenomic sequencing results, the top four microbiota communities at the phylum taxonomic level in groups D1 and D7 were selected, and the remaining were categorized as “others.” The results are presented as a relative abundance histogram. The vertical coordinate represents the sequence number of the anal swab sample (sample name), and the horizontal coordinate represents the relative abundance of the community.

To provide a comprehensive understanding of the gut microbiota of critically ill children, we analyzed the composition at each taxonomic level of colonization. At the phylum taxonomic level, *Bacteroidetes*, *Firmicutes*, *Proteobacteria*, and *Actinobacteria* were the main phyla in critically ill children before and after PICU treatment, with *Bacteroidetes* and *Firmicutes* dominating (Figures 3A,B). In a controlled analysis of two stool samples from all children, the relative abundances of both *Bacteroidetes* and *Actinobacteria* decreased, compared with those at PICU admission, while the relative abundances of *Firmicutes* and *Proteobacteria* increased; however, none of these changes were statistically different. Notably, the composition of the gut microbiota of critically ill children varied greatly between individuals and that the proportion of individual microbiota changed considerably after a short period of PICU treatment. For example, in the first child (S1), the sum of the relative abundances of *Bacteroidetes* in the anal swab sample on day 1 of PICU admission exceeded 92%, while the relative abundance of *Proteobacteria* on day 7 of admission was approximately 35%, and the relative abundance of *Bacteroidetes* was approximately zero (Figures 3A,B).

**Figure 4:**
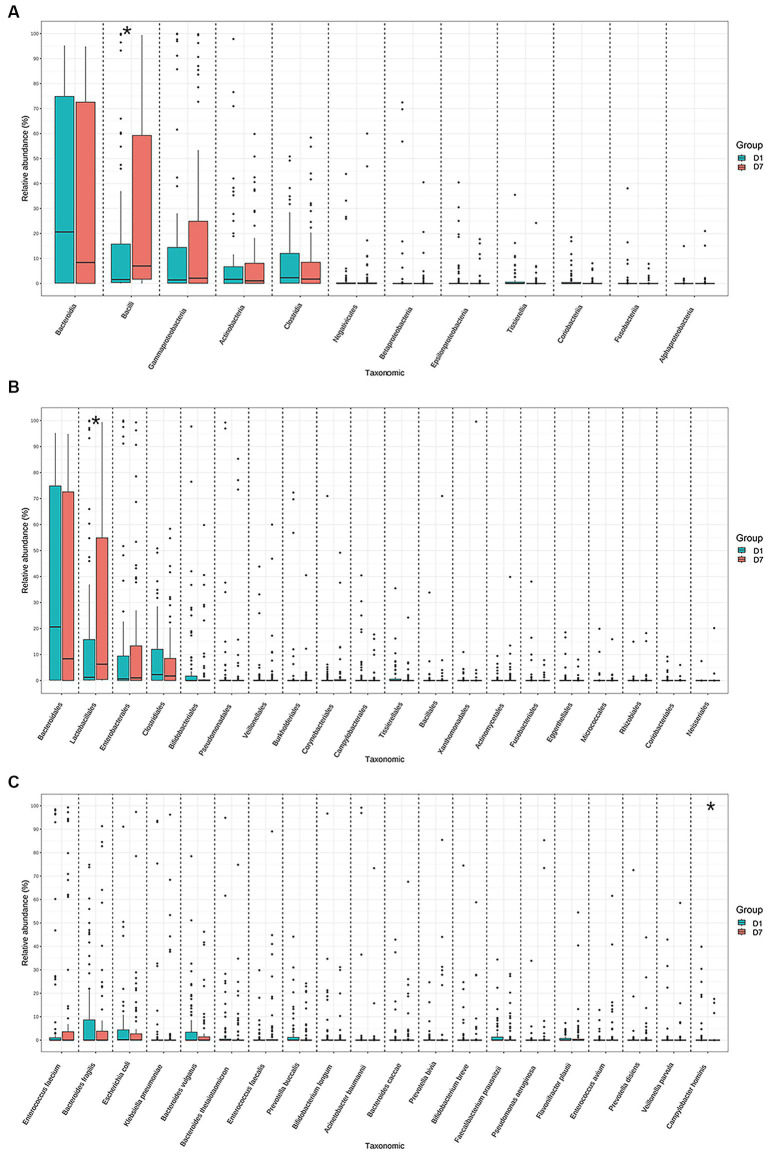
Relative abundances of the top 20 bacteria at the class, order, and species levels in the D1 and D7 groups. Distribution of the top 20 bacterial communities at the **(A)** class, **(B)** order, and **(C)** species levels. *p < 0.05.

Further controlled analyses of the gut microbiota of the children at the other levels were performed. The results showed that median and quartiles of both relative abundance (%) of *Bacilli* (15.79 [0.34, 19.42] vs. 70.03 [10.73, 59.56], *p* = 0.03) and *Lactobacillales* (1.25 [0.14, 19.41] vs. 6.29 [0.20, 58.41], *p* = 0.04) were significantly higher after a short period of PICU treatment in critically ill children. Whereas the mean ± standard deviation of relative abundance (%) of *Campylobacter hominis* was significantly lower (2.10 ± 0.85 vs. 0.63 ± 0.37, *p* = 0.02) (Figure 4; Supplementary Table S3).

**Figure 5:**
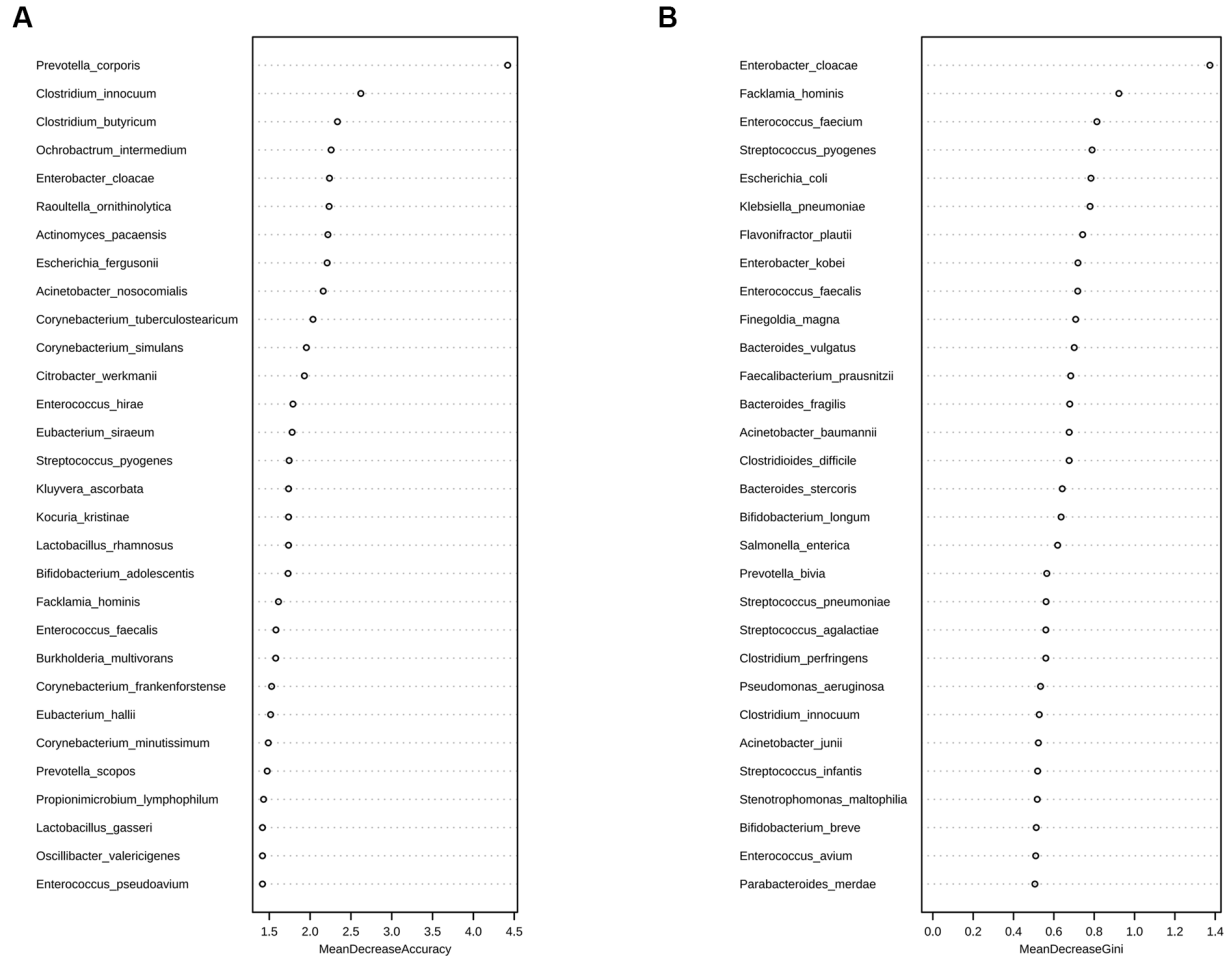
Random forest analysis of the D1 and D7 groups at the species level. Random forest analysis is a machine-learning algorithm that can effectively classify and predict grouped samples. The Accuracy **(A)** and Gini **(B)** indices are common evaluation metrics, with higher values indicating greater importance of the variable. The horizontal coordinates represent the values of Accuracy and Gini indices, and the vertical coordinates represent the strain names. The figure shows the strains that played major roles in the D1 and D7 groups, with *Prevotella coporis* and *Enterobacter cloacae* having the most significant classification effects.

A random forest analysis was also performed to analyze the possibility of using the gut microbiota as a novel biological marker for assessing changes in the condition of critically ill children at the species level. The assessment of Accuracy and Gini indices revealed that *Prevotella coporis* and *Enterobacter cloacae* played major roles in grouping as biological markers to distinguish the two groups (Figures 5A,B).

#### 3.3.3. Functional analysis of gene expression in the gut microbiome

**Figure 6:**
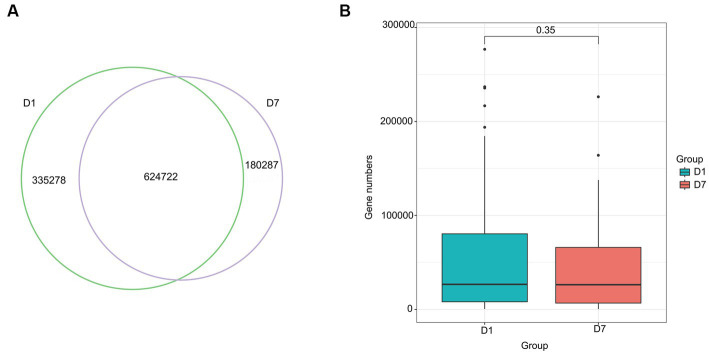
Venn diagram and Box diagram of the number of genes detected in the D1 and D7 groups. **(A)** Venn diagram of the number of unique or shared genes detected in the gut microbiota of the D1 and D7 groups. **(B)** Box plot of the number of genes detected in the gut microbiota of the D1 and D7 groups.

The Venn diagram in Figure 6A shows that 960,000 genes were detected in the gut microbiota of the D1 group and 805,009 ARG in the D7 group, of which, 624,722 genes were common to both groups. Although the number of genes decreased after 7 days of PICU treatment, compared with that at the time of admission to the PICU, this change was not statistically significant (*p* > 0.05) (Figure 6B).

**Figure 7:**
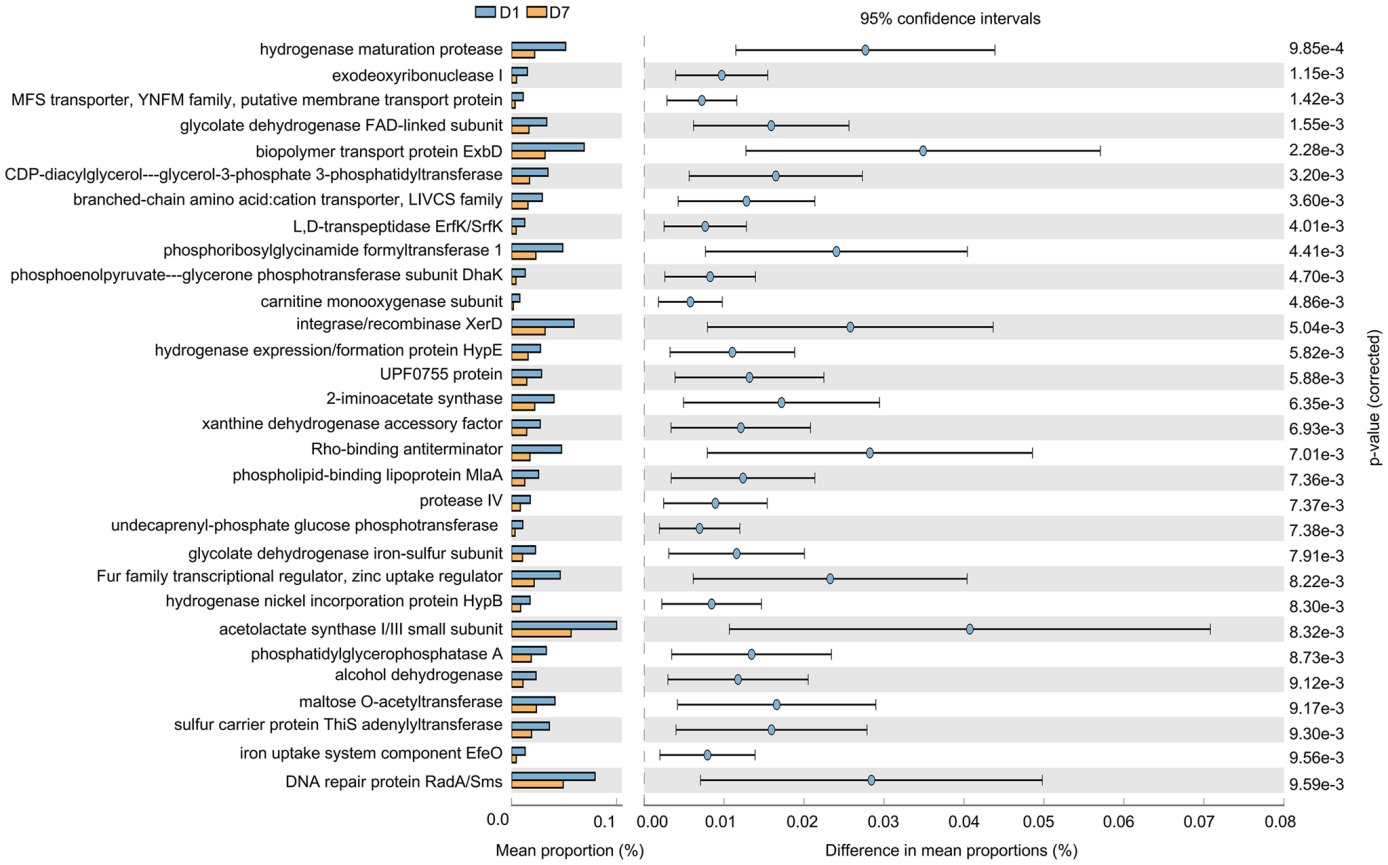
Gene function prediction of KEGG pathways. The histograms on the left represent the names of KEGG pathways and their relative abundances, and the dot plots on the right represent the corrected *p*-values. Corrected *p* < 0.05 was considered significant and retained.

As shown in Figure 7, the gut microbiota gene function in children changed significantly after a short-term PICU treatment. A total of 30 significantly altered KEGG pathways were obtained using *P* corrected <0.01 as the screening criterion, and the expression of these 30 pathways was suppressed. They mainly included changes in enzyme and transcription factor activities during metabolism, represented by phosphoribosylglycinamide formyltransferase 1, and alterations in enzyme and protein activities during DNA catabolism and repair, represented by DNA repair proteins RadA/Sms and exodeoxyribonuclease I. In addition, changes were observed in the biopolymer transport protein ExbD as a representative of substance transport protein activity. This suggests that the imbalance in intestinal microorganisms was accompanied by changes in the processes of material metabolism, material transport, and genetic material breakdown and repair.

**Figure 8:**
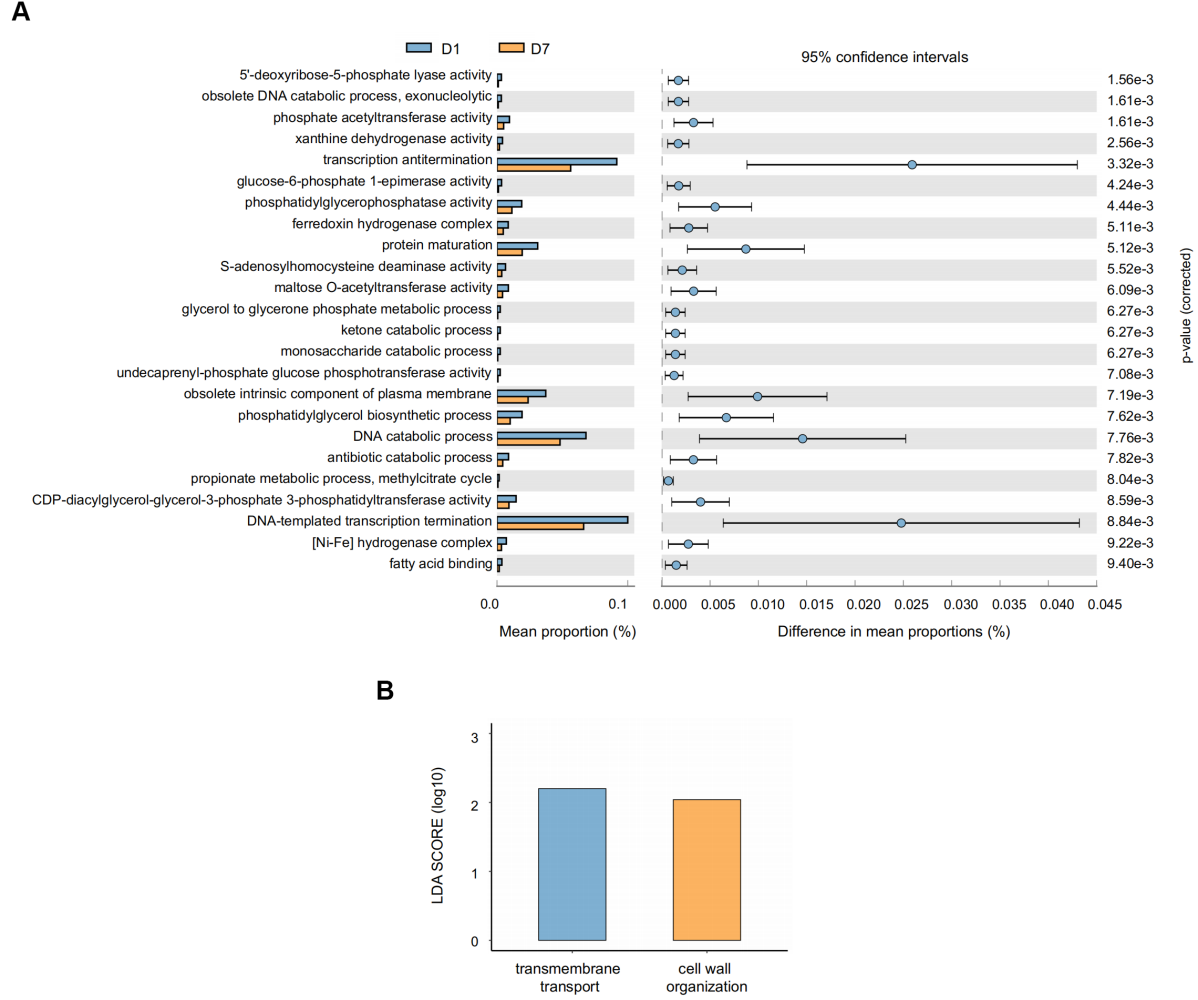
Gene function prediction and LEfSe analysis of GO entries. **(A)** The histograms on the left represent the names of GO entries and their relative abundances, and the dot plots on the right represent the corrected *p*-values. A corrected *p* < 0.05 was considered significant and retained. **(B)** The specific changed GO entries identified by linear discriminant analysis (LDA) and effect size (LEfSe) analysis were presented, and LDA scores >2.0 were considered significant.

Similarly, we comparatively analyzed the expression of GO entries by *P* corrected <0.01 (Figure 8A) and found that 24 GO entries underwent significant down-regulation after short-term PICU treatment. The main biological processes (BPs) involved included DNA-templated transcription termination, DNA catabolic process, and transcription antitermination; of them, the DNA catabolic process was similar to that found in the KEGG analysis of the exodeoxyribonuclease I activity. This was consistent with the changes in exodeoxyribonuclease I activity found in the KEGG analysis. Furthermore, LEfSe (Figure 8B) showed that two GO entries, GO:0071555 (cell wall organization) and GO:005508 (transmembrane transport), changed significantly after short-term PICU treatment. The involved BP of transmembrane transport was consistent with the changes in transporter protein activity in the KEGG analysis.

#### 3.3.4. Differential analysis of the expression of antimicrobial resistance genes in the gut microbiome

**Figure 9:**
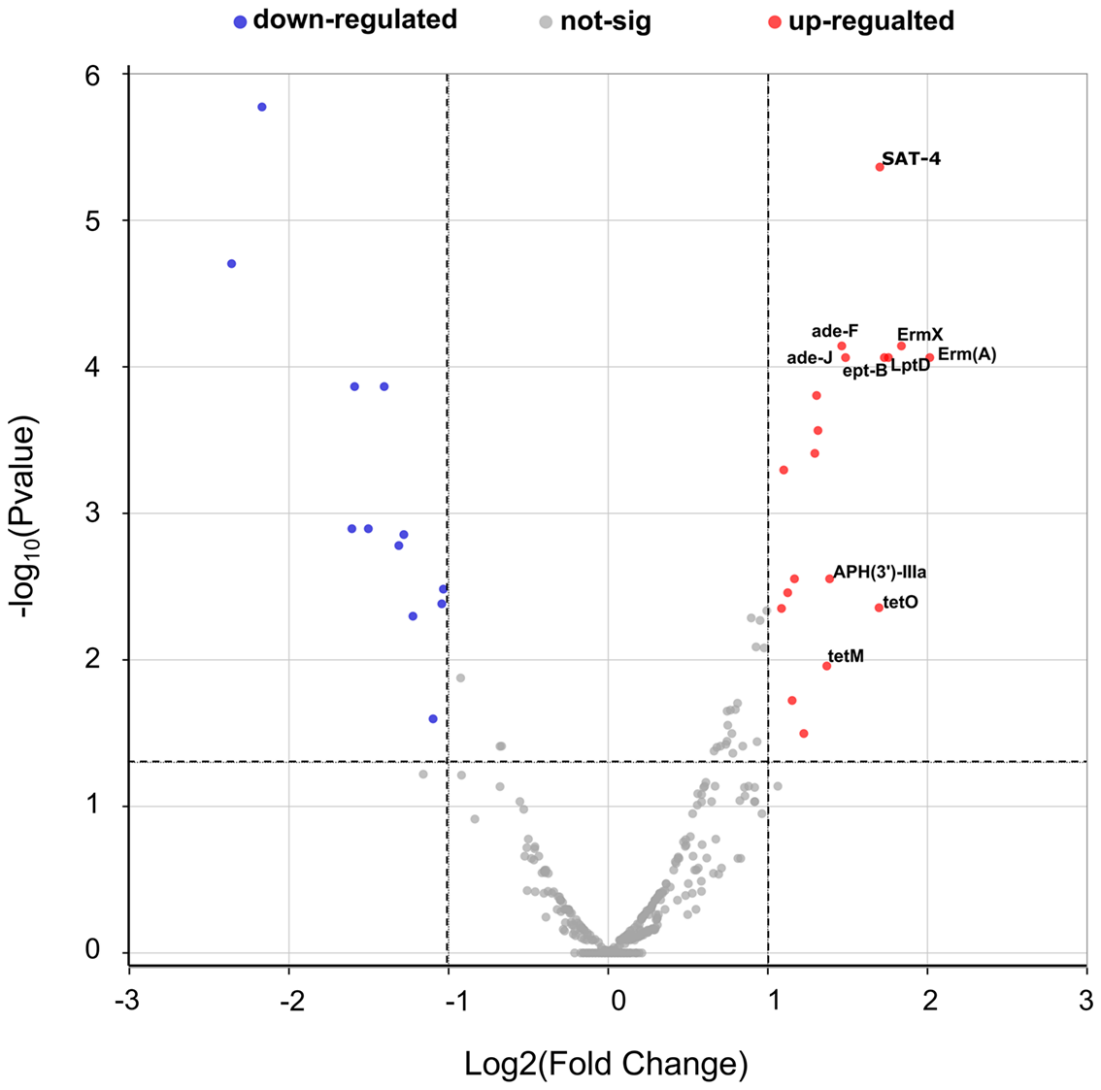
Volcano plots of antimicrobial resistance gene expression in the D1 and D7 groups. The horizontal coordinates in the graph represent the log_2_ fold change, and the vertical coordinates represent the-log10 (*p*-value). The horizontal dashed line in the figure represents the *p*-value threshold (0.05), and the two vertical dashed lines represent log_2_FC = 1/−1. The red dots represent *p* < 0.05 and log_2_ fold change ≥1, which means that the expression of resistance genes is significantly upregulated. The blue dots represent *p* < 0.05 and log_2_ fold change ≤ − 1, which means that the expression of resistance genes is significantly down-regulated. While the gray dots mean that there is no significant change. The top ten up-regulated resistance genes are labeled in the figure.

Overall, 31 antibiotic resistance genes (ARGs) with significant differences were detected in the D7 group compared with the D1 group, including 19 resistance genes with upregulated expression. The top 10 differentially up-regulated resistance genes, in order, were: Erm(A), ErmX, LptD, eptB, SAT-4, tetO, adeJ, adeF, APH(3′)-IIIa, and tetM (Figure 9; Supplementary Table S4). The Comprehensive Antibiotic Resistance Database (CARD) showed that tetM, LptD, and eptB can be found in resistant *Klebsiella pneumoniae*, and LptD can cause resistance to carbapenem antibiotics. Besides, adeJ, adeF, and tetM can be found in resistant *Acinetobacter baumannii*, and adej can cause resistance to carbapenem antibiotics.

## 4. Discussion

The human gastrointestinal tract stores a large number of microorganisms, and close communication occurs between them and their hosts. Disturbances in the structure and function of gut microbiota are associated with numerous pathological processes in the human body, including inflammatory bowel disease, type II diabetes, and colorectal cancer (Manor et al., 2020). In recent years, with increasing research, more studies have demonstrated that the gut is a key factor in the initiation and development of critical illnesses and that intestinal failure is associated with poor prognosis (Szychowiak et al., 2022). Therefore, understanding the structure and function of gut microbiota and their role in critical illnesses is crucial.

In the present study, children received broad-spectrum antibiotics intravenously after enrollment, and different proportions of children received proton pump inhibitors, vasoactive drugs, invasive mechanical ventilation, parenteral nutritional support, and probiotics, in addition to exhibiting varying conditions such as underlying disease, site of infection, pathogenic bacteria, and immune status. The microbial environment in the gut of critically ill patients, often influenced by genetics, diseases, and therapeutic factors, is often disturbed (Wozniak et al., 2022), characterized by reduced abundance and diversity and an increase in conditionally pathogenic bacteria (e.g., *Clostridium difficile*, multi-drug-resistant bacteria) (Szychowiak et al., 2022). For example, intestinal emptying can be reduced in critically ill patients with decreased bacterial excretion, leading to excessive growth of pathogenic bacteria in the intestine (Dickson, 2016; Ladopoulos et al., 2018). Antibiotic use has been associated with decreased gut microbiota diversity (Ramirez et al., 2020). In addition, a study in adults showed that the combination of meropenem, gentamicin, and vancomycin led to an increase in *Enterobacter* and other pathogenic bacteria in the intestine, whereas *Bifidobacteria* and butyrate-producing flora were reduced (Palleja et al., 2018). Drugs such as non-steroidal anti-inflammatory drugs, proton pump inhibitors, and β-blockers can affect bacterial growth by altering the pH of the intestine (Wozniak et al., 2022), and continuous parenteral nutrition has been associated with significant disturbances in the flora (Dahlgren et al., 2019).

In this study, the diversity of the gut microbiota was investigated. The ACE and Chao1 indices of the children in the D7 group were significantly lower than those in the D1 group. The ACE and Chao1 indices are two indicators that describe the richness of the gut microbiota (Szychowiak et al., 2022), which implies that a significant decrease in the number of bacterial species in the gut microbiota occurred after 7 days of treatment in the PICU. While the Shannon and Simpson indices, describing both richness and evenness (Szychowiak et al., 2022), did not change significantly in our study.

Studies performed on the gut flora of critically ill children, using follow-up data, remain insufficient. Rogers et al. found that the Shannon diversity index of the gut flora was decreased significantly compared with healthy children, and it was significantly negatively correlated with the time spent in the PICU (Rogers et al., 2016). This differed from our results, which showed that the Shannon index did not change significantly after short-term PICU treatment. One of the reasons for this discrepancy might be that we followed-up with the patients for different periods of time. Our study further investigated changes in the composition of the gut microbiota in critically ill children after short-term PICU treatment, while Rogers et al. followed-up with patients for 30 days. The results showed that on both days 1 and 7 of PICU admission, the composition of the children’s gut microbiota at the phylum level was dominated by the *Firmicutes*, *Bacteroides*, *Aspergillus*, and *Actinomycetes*, and a majority of patients had a predominant proportion of the *Firmicutes* and *Bacteroides*. In 2005, Eckburg et al. found that the sum of the relative abundances of *Firmicutes* and *Bacteroidetes* exceeded 90% in the human gut microbiota (Eckburg et al., 2005). We further compared changes in flora at the phylum level, where the relative abundance of *Bacillus* decreased. β-hexosaminidase in the *Methanobacterium* has been shown to be a driver of lymphocyte-specific differentiation and that β-hexosaminidase-specific lymphocytes are protective against intestinal inflammation in a mouse model of colitis (Bousbaine et al., 2022). A study conducted on 115 critically ill patients revealed similar results, with patients leaving the ICU showing a significant decrease in the relative abundance of the *Firmicutes* and *Bacteroidetes* of the gut microbiota, a significant increase in the *Proteobacteria*, and an increase in pathogenic colonization with *Enterobacter* spp. and *Staphylococcus* spp., compared with those at the time of admission to the ICU (Mcdonald et al., 2016).

Notably, there were differences in the composition of the flora among individual patients after ICU treatment (Figure 3). Ojima et al. (2016) conducted a longitudinal observational study of the gut microbiota of 12 mechanically ventilated patients in a large tertiary care hospital; they found that the percentages of the *Bacteroidetes* and *Firmicutes* changed significantly during the follow-up period and that an extreme imbalance of flora might be associated with poor patient prognosis. Disturbances in the flora have been shown to be associated with increased susceptibility to nosocomial infections, sepsis, organ failure, and severe COVID-19 (Lynch and Pedersen, 2016; Ojima et al., 2016; Yeoh et al., 2021). In the present study, children treated in the PICU showed significant changes in C-reactive protein, calcitonin, white blood cells, platelets, creatinine, and lactate levels. In addition, the relative abundance of colonies such as *Bacilli*, *Lactobacillales,* and *Campylobacter hominis* changed significantly. Chao1 and ACE indices also decreased significantly, and the random forest analysis revealed that the species levels of *Prevotella corporis* and *Enterbacter cloacae* were specific markers before and after PICU treatment in critically ill children. This suggests that future studies should correlate changes in the gut microbiota of patients with changes in clinical indicators to provide novel reference indicators for assisting in the assessment of changes in patient conditions.

Our study found that after a short period of PICU treatment, the gene function of the gut microbiota of critically ill children can undergo significant changes, mainly including the processes of metabolism, DNA degradation, and transmembrane transport. However, it should be noted that our results are derived from computerized predictions, and their exact roles in human physiological activities remain unclear. Nevertheless, several studies have confirmed the involvement of metabolites of the gut microbiota in the regulation of vital organ functions. Hayakawa et al. (2011) found that changes in the composition of the flora, including increases in *Enterococcus faecalis* and *Pseudomonas aeruginosa*, accompanied by decreases in the three major short-chain fatty acids, butyric acid, propionic acid, and acetate, could occur within 6 h of the onset of critical illness. Short-chain fatty acids are products of the glycolytic process of dietary fibers by intestinal microorganisms, including acetate and butyrate; they are involved in regulating human immune functions, including promoting the differentiation and expansion of T cells, forming a complete mucosal immune system, and influencing the phagocytosis of macrophages (Furusawa et al., 2013). Butyrate also plays a role in regulating the transcription factor HIF-1, which is involved in maintaining the stable function of the intestinal barrier by decreasing the oxygen concentration in tissues (Lopez-Siles et al., 2017). Fatty acids may also be involved in the physiological functions of the brain, lungs, and cardiovascular system by activating the vagus nerve, reducing lung inflammation, and affecting renin secretion (Wozniak et al., 2022). Short-chain fatty acid levels have been shown to be negatively correlated with the severity of portal hypertension and systemic inflammation, emphasizing the role of gut microbiota in enterohepatic interactions and the progression of liver pathologies such as cirrhosis (Juanola et al., 2019). Short-chain fatty acids also play a role in acute kidney injury (AKI) by modulating the inflammatory response, and thus improving AKI outcomes (Andrade-Oliveira et al., 2015). Therefore, gut microbiota disorders can cause a decrease in beneficial metabolites, further causing adverse outcomes such as immune and organ dysfunction and increasing susceptibility to disease. Moreover, GO analysis in our study revealed that bacterial cell wall synthesis and the transmembrane transport of macromolecules were affected in the gut microbiota of critically ill children after a short period of ICU treatment. We hypothesized that this may be related to patients receiving antibiotics, proton pump inhibitors, and inadequate organ perfusion, causing a disturbance of the intestinal microenvironment and further affecting the physiological activity of the flora.

The intestine is a major site of drug-resistant bacteria. A healthy gut microbiota is a stable and diverse community that protects the host from invasion by pathogenic bacteria. Antibiotics can disrupt the stable ecosystem of the gut, providing conditions for colonization by drug-resistant bacteria, increasing resistance gene load, and further spreading resistant bacteria to other sites, causing infection (Anthony et al., 2021). Norgaard et al. found that the use of broad-spectrum β-lactam antibiotics was most significantly associated with increased microbial destruction and resistance characteristics in patients treated with hematopoietic stem cell transplantation (Nørgaard et al., 2023). The increasing prevalence of carbapenem-resistant *Enterobacteriaceae* poses a major global health threat (Jean et al., 2022). Studies have shown that the risk of infection with carbapenem-resistant *Acinetobacter baumannii* can increase by four-fold with exposure to carbapenems, and a new meta-analysis confirmed an association between carbapenem-resistant *P. aeruginosa* and increased mortality (Brink, 2019).

Our study has some limitations. First, while the purpose of this study was to describe the composition and function of the flora of critically ill children after PICU treatment, the degree of influence of factors was not evaluated. Second, owing to the observational nature of our study, it was not possible to control for the variables that might have affected the intestinal flora. Third, this study was a single-center study, and there was a certain selection bias.

## 5. Conclusion

After short-term treatment in the PICU, the richness of the gut microbiota in critically ill children was significantly decreased, while the bacterial diversity and the community structure between groups remained stable to some extent. The composition of some colonies was also altered significantly, with a significant increase in the relative abundances of *Bacilli* and *Lactobacillales* and a significant decrease in the relative abundance of *C. hominis*. GO and KEGG analyses showed that gene functions of the gut microbiota were also altered, mainly in genes responsible for metabolism, DNA catabolism, and transmembrane transport. In addition, the expression of resistance genes in critically ill children was changed significantly after short-term treatment in the PICU. The top 10 up-regulated genes were *Erm(A)*, *ErmX*, *LptD*, *eptB*, *SAT-4*, *tetO*, *adeJ*, *adeF*, *APH(3′)-IIIa*, and *tetM*.

## Data availability statement

The data presented in this study are deposited in the NCBI SRA repository, accession number PRJNA1033539 (http://www.ncbi.nlm.nih.gov/bioproject/1033539).

## Ethics statement

The studies involving humans were approved by Ethics Committee of Xinhua Hospital, Shanghai Jiao Tong University School of Medicine. The studies were conducted in accordance with the local legislation and institutional requirements. Written informed consent for participation in this study was provided by the participants’ legal guardians/next of kin.

## Author contributions

JX and YZ: study design. HM and YZ: data collection. JX and XK: statistical analysis. YX: data interpretation. JX, YX, and YZ: manuscript preparation. JL and JX: literature search. All authors contributed to the article and approved the submitted version.

## Footnotes

^1^Download from https://www.ncbi.nlm.nih.gov/.

^2^https://gmrepo.humangut.info/home

^3^http://www.geneontology.org

^4^http://www.genome.jp/kegg/
